# 16S rRNA Sequencing Analysis Uncovers Dose-Dependent Cupric Chloride Effects on Silkworm Gut Microbiome Composition and Diversity

**DOI:** 10.3390/ani14243634

**Published:** 2024-12-17

**Authors:** Wantao Rong, Yanqi Wei, Yazhen Chen, Lida Huang, Shuiwang Huang, Yiwei Lv, Delong Guan, Xiaodong Li

**Affiliations:** 1 Guangxi Key Laboratory of Sericulture Ecology and Applied Intelligent Technology, Hechi University, Hechi 546399, China; 18023@hcnu.edu.cn (W.R.); 2020101536@hcnu.edu.cn (Y.W.); 2020660058@hcnu.edu.cn (Y.C.); 2020101520@hcnu.edu.cn (L.H.); 2020101505@hcnu.edu.cn (S.H.); 2020101530@hcnu.edu.cn (Y.L.); 2 Guangxi Collaborative Innovation Center of Modern Sericulture and Silk, Hechi University, Hechi 546399, China

**Keywords:** silkworm, gut microbiome, cupric chloride exposure, heavy metal safety, 16S rRNA sequencing, microbial balance

## Abstract

Copper-based pesticides are extensively used in agriculture, yet their impacts on beneficial insects remain poorly understood. Here, we investigate how cupric chloride exposure affects the gut microbiome of Bombyx mori, a model organism crucial for silk production. Using 16S rRNA sequencing, we analyzed the gut bacterial communities of fifth-instar silkworm larvae exposed to different concentrations of cupric chloride (0, 4, and 8 g/kg) in an artificial diet. The high-dose exposure dramatically altered the microbial diversity and community structure, where the Bacteroidota abundance decreased from 50.43% to 23.50%, while Firmicutes increased from 0.93% to 18.92%. A network analysis revealed complex interactions between the bacterial genera, with Proteobacteria and Firmicutes emerging as key players in the community response to copper stress. The functional prediction indicated significant shifts in metabolic pathways and genetic information processing in the high-dose group. Notably, the low-dose treatment induced minimal changes in both the taxonomic composition and predicted functions, suggesting a threshold effect in the microbiome response to copper exposure. Our findings provide novel insights into how agricultural chemicals influence insect gut microbiota and highlight potential implications for silkworm health and silk production. This work contributes to understanding the ecological impacts of copper-based pesticides and may inform evidence-based policies for their use in sericulture regions.

## Simple Summary

Copper-based pesticides are widely used in agriculture, but their impact on beneficial insects, like silkworms, remains unclear. This study investigated how cupric chloride affects the gut bacteria of silkworms, which are crucial for silk production. We fed silkworms an artificial diet that contained different amounts of cupric chloride and analyzed the bacteria in their guts. Interestingly, low levels of copper had a minimal impact, suggesting the silkworms could tolerate the common use of copper compounds. Only a high level of copper changed the types and amounts of bacteria present. Some bacteria decreased, while others, particularly those that can tolerate harsh conditions, became more common. These changes also affected the potential functions of the gut bacteria community. Our findings provide new insights into the safe threshold on agricultural chemicals affect beneficial insects indirectly through their gut bacteria. This knowledge could help develop better practices for using copper-based pesticides in areas where silkworm farming is important, balancing crop protection with the health of these valuable insects.

## 1. Introduction

Copper-based compounds have long been integral to agricultural practices, serving dual roles as both essential micronutrients and potent antimicrobial agents [1,2,3]. Among these, cupric chloride (CuCl^2^) has emerged as a particularly versatile compound, finding widespread application in animal husbandry as a feed additive and in crop protection as an effective fungicide and bactericide [4,5,6]. Parallel to its nutritional applications, cupric chloride has established itself as an indispensable tool in the agriculturalist’s arsenal against plant diseases [7]. As a broad-spectrum fungicide and bactericide, it offers protection against a wide array of phytopathogens, including those responsible for devastating crop losses in economically important species, such as apple [8]. However, despite the well-documented benefits and widespread adoption of cupric chloride in global agriculture, its use has not been without controversy. Concerns regarding potential environmental accumulation and non-target effects have led to increased scrutiny and, in some cases, restrictive policies on its application [3,9,10]. One such example is the apprehension surrounding the use of copper-based pesticides in sericulture regions, particularly in China, where the mulberry silkworm (*Bombyx mori*) plays a crucial economic and cultural role [11,12].

The silk industry in China, which accounts for over 80% of global silk production, has traditionally adopted a cautious stance toward the application of copper-based pesticides on mulberry trees (*Morus alba*), the sole food source for *B. mori* [13,14]. This caution stems from a prevailing belief that cupric chloride pesticides might pose a serious threat to silkworm health and silk quality [15]. However, it is noteworthy that this perception appears to be largely based on precautionary principles rather than robust scientific evidence. The actual impact of cupric chloride on silkworm physiology, development, and silk production remains poorly understood, with limited empirical data to support or refute these concerns. Even in some cases, copper-compounds were reported to enhance the quality of silk fibers, although slightly inhibit the larva development [16,17]. This knowledge controversy presents a critical challenge for the sericulture industry, potentially hindering the adoption of effective pest management strategies and compromising mulberry crop yields. There is an urgent need for comprehensive research to elucidate the true nature and extent of cupric chloride’s impact on *B. mori*, particularly with respect to potential sublethal effects that could influence silk production and quality.

Recent advances in molecular biology and high-throughput sequencing technologies have opened new avenues for investigating the subtle effects of environmental stressors on organism health. Of particular interest is the growing recognition of the gut microbiome as a critical mediator of host–environment interactions. The gut microbiota plays essential roles in nutrition, immunity, and overall physiology across diverse animal taxa, including insects [18,19,20]. In *B. mori*, the gut microbiome was shown to influence various aspects of silkworm biology, including digestion, immunity, and even silk production [21,22]. Given the potential for agricultural chemicals to perturb microbial communities, an examination of cupric chloride’s effects on the silkworm gut microbiome presents a promising approach to assess its impact on silkworm health.

In light of these considerations, the present study aimed to conduct a comprehensive assessment of the effects of cupric chloride on the gut microbiota of *B. mori* using 16S rDNA sequencing. While the typical acute oral LD50 of cupric chloride for insects is around 0.11 g/kg [23], we deliberately chose to employ higher concentrations (4 g/kg and 8 g/kg) for several key reasons: First, copper compounds can accumulate in agricultural soils over years of repeated applications, potentially reaching elevated levels in mulberry leaves. Previous studies have reported copper accumulation in agricultural soils can be up to a very high concentration of 1700 mg/kg after the long-term usage of copper-based compounds [24]. Second, these concentrations allowed us to establish clear dose–response relationships and identify potential threshold levels for adverse effects on the gut microbiome. Third, by using these elevated doses in a controlled laboratory setting, we could better understand the resilience and adaptation mechanisms of the silkworm gut microbiome under extreme metal stress conditions. While these concentrations exceed typical environmental levels, they provide valuable insights into the upper boundaries of microbial community responses to copper exposure. This knowledge can inform risk assessments for more environmentally relevant exposure scenarios.

By employing such high doses, we aimed to establish clear upper boundaries for cupric chloride toxicity, providing a comprehensive framework for assessing risks associated with more realistic exposure levels. This approach will not only contribute to our understanding of cupric chloride’s specific effects on *B. mori* but also offer broader insights into the interactions between agricultural chemicals and insect gut microbiomes.

## 2. Materials and Methods

### 2.1. Silkworm Rearing and Experimental Design

*Bombyx mori* larvae (Liangguang No. 2 strain) were obtained from Hechi University, China. The silkworms were reared under standard conditions (25 ± 1 °C, 70 ± 5% relative humidity, 8 h light/16 h dark cycle) in a dedicated silkworm-rearing facility. The larvae were fed an artificial diet ad libitum throughout the experiment, except where noted. The diet consisted primarily of soybean meal (45%) supplemented with corn starch (25%); wheat bran (15%); mulberry leaf powder (10%); and other essential ingredients, including vitamin and mineral premix (3%), citric acid (1%), and preservatives (1%). Three experimental groups were established: control group (CMK)—standard diet without cupric chloride supplementation; low-dose group (CMF)—diet supplemented with 4 g/kg cupric chloride; high-dose group (CME)—diet supplemented with 8 g/kg cupric chloride. Cupric chloride (CuCl^2^·2H_2_O, ≥99% purity, DAMAO—Chemical reagent factory, Dongli District, Tianjing, China) was thoroughly mixed into the finely ground artificial diet. The supplemented artificial diets were steamed for one hour and then kept ready for use. The control group was treated similarly but without the cupric chloride addition. The fifth-instar larvae of the silkworm were randomly divided into three groups, with 50 larvae in each group, and fed with diets that contained different concentrations of Cu^2+^. Fifth-instar larvae were used for all experiments, with seven biological replicates per treatment group. Each replicate consisted of 3 individual silkworms. The experiment was conducted for 96 h, after which the larvae were subjected to a 24 h starvation period, followed by dissection of the gut and analysis of the microbiome.

### 2.2. Gut Dissection and Microbial DNA Extraction

Silkworm larvae were surface-sterilized with 75% ethanol and dissected aseptically. The entire digestive tract was removed, and the midgut region was isolated. Midgut contents from the 3 individuals in each replicate were pooled and homogenized in sterile phosphate-buffered saline (PBS, pH 7.4). Total genomic DNA was extracted from the homogenized gut samples using the QIAamp PowerFecal Pro DNA Kit (QIAGEN, Hilden, Germany) following the manufacturer’s instructions. DNA quantity and quality were assessed using a NanoDrop 2000 spectrophotometer (Thermo Fisher Scientific, Waltham, MA, USA) and 1% agarose gel electrophoresis.

### 2.3. 16S rRNA Gene Amplification and Sequencing

The V4–V5 hypervariable regions of the bacterial 16S rRNA gene were amplified using the universal primers 515F (5′-GTGCCAGCMGCCGCGGTAA-3′) and 806R (5′-GGACTACHVGGGTWTCTAAT-3′). Each primer was tagged with a unique barcode sequence to allow for sample multiplexing. PCR amplification was performed in 30 μL reactions containing 15 μL of Phusion High-Fidelity PCR Master Mix (New England Biolabs, Ipswich, MA, USA), 0.2 μM of each primer, and 10 ng of template DNA. The PCR cycling conditions were as follows: initial denaturation at 98 °C for 1 min; 30 cycles of 98 °C for 10 s, 50 °C for 30 s, and 72 °C for 30 s; and a final extension at 72 °C for 5 min. The PCR products were purified using Agencourt AMPure XP beads (Beckman Coulter, Brea, CA, USA) and quantified using the Qubit dsDNA HS Assay Kit (Invitrogen, Carlsbad, CA, USA). Equal amounts of purified amplicons were pooled for a sequencing library preparation using the TruSeq DNA PCR-Free Library Preparation Kit (Illumina, San Diego, CA, USA). The prepared libraries were sequenced on an Illumina NovaSeq 6000 platform (Illumina, San Diego, CA, USA) using 2 × 250 bp paired-end sequencing chemistry.

### 2.4. Bioinformatic Analysis

The raw sequencing data were processed using QIIME 2 version 2021.4 [25]. Briefly, demultiplexed paired-end reads were quality filtered, denoised, and merged using DADA2 [26] with default parameters. Chimeric sequences were identified and removed using the consensus method in DADA2. Amplicon Sequence Variants (ASVs) were aligned using MAFFT v7.475 [27] and used to construct a phylogenetic tree with FastTree 2 [28]. The taxonomy was assigned to ASVs using the q2-feature-classifier [29] against the SILVA 138.1 SSU rRNA database [30]. Alpha diversity metrics (Observed ASVs, Shannon index, Simpson index, Chao1, ACE, Good’s coverage, and Faith’s Phylogenetic Diversity) were calculated using the q2-diversity plugin in QIIME 2 [25]. The beta diversity was assessed using weighted and unweighted UniFrac distances. Principal Coordinate Analysis (PCoA) and Principal Component Analysis (PCA) were performed using the phyloseq R package v1.36.0 [31]. PERMANOVA was used to test for significant differences in community composition between groups. Functional predictions were made using PICRUSt2 v2.4.1 [32] with default parameters. Predicted MetaCyc pathways were analyzed for differential abundance using STAMP v2.1.3 [33]. All statistical analyses and plots were generated using the online genecloud platform (https://www.genescloud.cn/, accessed on 3 September 2024).

## 3. Results

### 3.1. Effects of Cupric Chloride Exposure on Silkworm Gut Microbiome Diversity

Exposure to cupric chloride induced significant alterations in the alpha diversity of the silkworm gut microbiome (Table 1 and Table S1). The control group (CMK) consistently exhibited the highest diversity across all measured indices. A dose-dependent decrease in microbial diversity was observed, where the high-dose group (CME) showed the most pronounced reduction. The number of observed Amplicon Sequence Variants (ASVs) decreased significantly from 235.14 ± 12.45 in CMK to 198.57 ± 9.83 in CMF, and further to 167.29 ± 8.61 in CME (*p* < 0.001). This trend was mirrored in other alpha diversity metrics, including the Shannon index (CMK: 3.76 ± 0.15, CMF: 3.24 ± 0.11, CME: 2.87 ± 0.09, *p* < 0.001) and Simpson index (CMK: 0.95 ± 0.01, CMF: 0.91 ± 0.01, CME: 0.88 ± 0.01, *p* < 0.01). The Chao1 and ACE estimators, which account for community richness, also showed significant decreases in the copper-treated groups (*p* < 0.001 for both indices). Notably, while Good’s coverage remained high across all groups (>0.99), indicating adequate sequencing depth, Faith’s Phylogenetic Diversity decreased markedly with the copper exposure (CMK: 21.63 ± 1.08, CMF: 18.41 ± 0.92, CME: 15.79 ± 0.79, *p* < 0.001), suggesting a reduction in the phylogenetic diversity.

**Figure 1:**
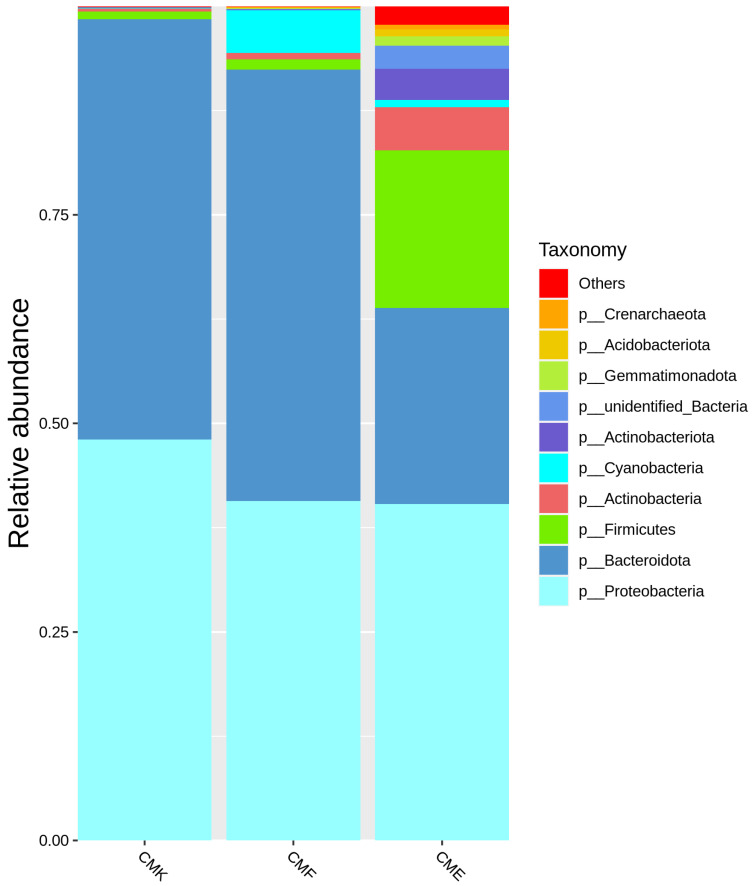
Relative abundance of bacterial phyla in the silkworm gut microbiome under different cupric chloride exposure conditions. The stacked bar plot shows the taxonomic composition at the phylum level for the control group (CMK), low-dose cupric chloride group (CMF, 4 g/kg), and high-dose cupric chloride group (CME, 8 g/kg). Each bar represents the mean relative abundance of the bacterial phyla across the replicates (*n* = 7). The phyla with relative abundances less than 1% in all groups are combined into the “Others” category.

The taxonomic composition of the silkworm gut microbiome underwent substantial shifts in response to cupric chloride exposure (Figure 1, Table S2). At the phylum level, Proteobacteria and Bacteroidota dominated across all the treatment groups, but their relative abundances were differentially affected by the copper exposure. In the control group (CMK), Bacteroidota was the most abundant phylum (50.43%), followed closely by Proteobacteria (48.03%). However, in the high-dose copper group (CME), the relative abundance of Bacteroidota decreased substantially to 23.50%, while the Proteobacteria remained relatively stable at 40.31%. Intriguingly, the Firmicutes phylum exhibited a marked increase in relative abundance with increased copper concentration, where it rose from 0.93% in the control group to 1.21% in the low-dose group (CMF), and further to 18.92% in the high-dose group (CME). This suggests that members of the Firmicutes phylum may possess higher tolerance to copper stress or the ability to capitalize on the altered gut environment. Several low-abundance phyla also demonstrated notable changes.

Actinobacteria increased from 0.36% in the control to 5.18% in the high-dose group. Cyanobacteria showed a substantial increase in the low-dose group (5.15%) compared with the control (0.08%) but decreased in the high-dose group (0.87%), indicating a non-linear response to copper exposure. Phyla such as Crenarchaeota, Acidobacteriota, and Gemmatimonadota, while remaining minor components of the community, showed consistent increases in relative abundance with increased copper exposure. Notably, the proportion of unidentified Bacteria increased with the copper exposure, from less than 0.001% in the control to 2.79% in the high-dose group, suggesting that copper stress may select for less well-characterized bacterial taxa. The “Others” category, which represented rare and unclassified phyla, similarly increased from 0.10% in the control to 2.20% in the high-dose group, further indicating a shift toward a more diverse array of minor taxa under copper stress.

**Figure 2:**
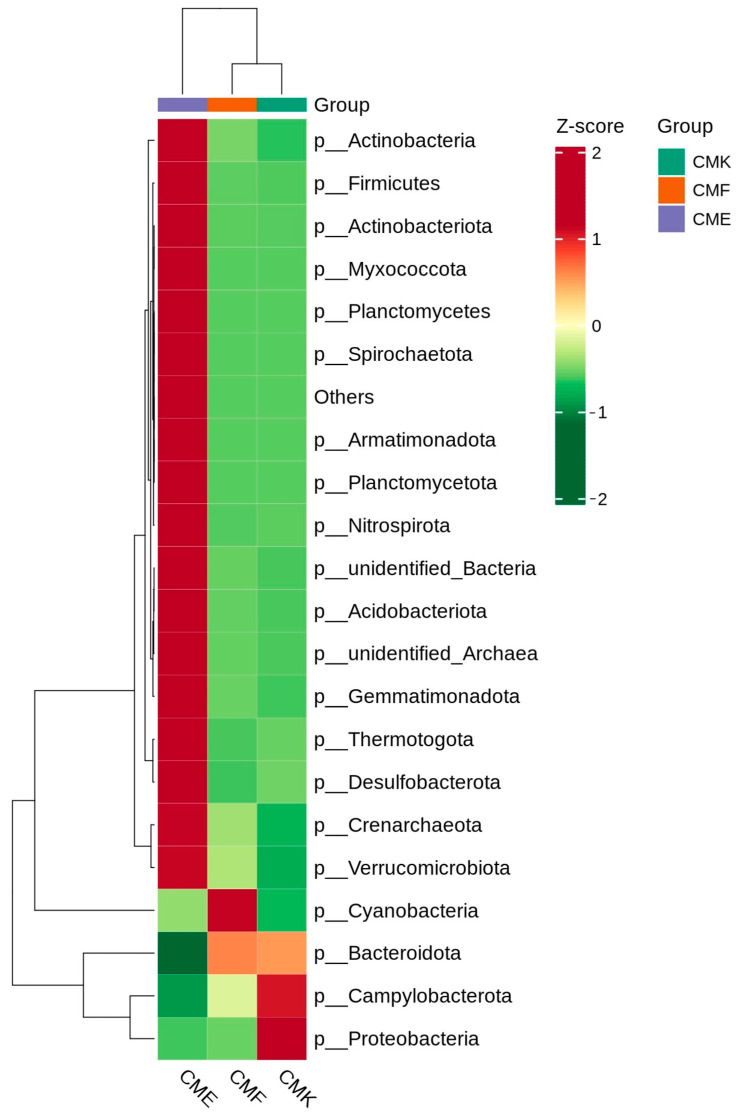
Heatmap showing the relative abundances of bacterial phyla in the silkworm gut microbiome under different cupric chloride exposure conditions. Each column represents a treatment group, and each row represents a bacterial phylum. The color scale ranges from green (lower abundance) to red (higher abundance), with white indicating values close to the mean. Hierarchical clustering was performed on both rows and columns using the Euclidean distance and complete linkage method.

Hierarchical clustering analysis revealed distinct patterns of bacterial phyla abundance across the three treatment groups (Figure 2, Table S3). The high-dose cupric chloride group (CME) exhibited the most pronounced alterations compared with the control (CMK) and low-dose (CMF) groups. In the CME group, several phyla demonstrated markedly increased abundances, as indicated by their positive Z-scores. These included Actinobacteria; Firmicutes; Actinobacteriota; Myxococcota; and members of less abundant phyla, such as Planctomycetes, Spirochaetota, and Armatimonadota. Concurrently, the CME group showed a significant decrease in the abundance of Bacteroidota and Proteobacteria, two of the most dominant phyla in the silkworm gut microbiome. The Cyanobacteria phylum displayed a unique pattern, with the highest abundance observed in the CMF group, while being less abundant in both the CME and CMK groups. This non-linear response to copper exposure suggests a complex interaction between Cyanobacteria and copper levels in the gut environment. The CMK group was characterized by the highest abundance of Proteobacteria and Campylobacterota, indicating that these phyla may be more sensitive to copper exposure.

**Figure 3:**
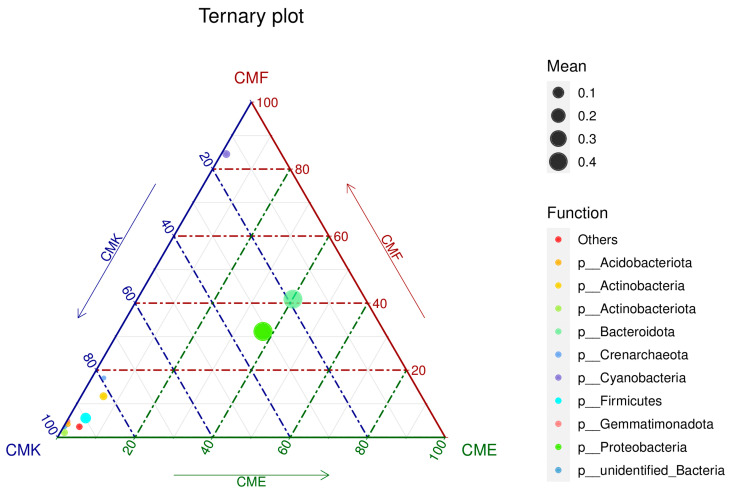
Ternary plot illustrating the differential distribution of dominant bacterial phyla in the silkworm gut microbiome under cupric chloride exposure.

To visualize the differences in dominant bacterial phyla between the three treatment groups, a ternary plot was constructed using the top 10 most abundant phyla (Figure 3). This plot provides a clear representation of the relative abundance and distribution of the key bacterial groups in response to the cupric chloride exposure. The ternary plot revealed distinct patterns of phylum-level abundance across the CMK (control), CMF (low-dose), and CME (high-dose) groups. Proteobacteria and Bacteroidota, represented by the largest circles, were the most abundant phyla across all the treatments. However, their distribution patterns differed markedly. Proteobacteria showed a slight bias toward the CMK group, indicating a higher relative abundance in the control condition. In contrast, Bacteroidota appeared more evenly distributed between the three groups, suggesting a more stable presence across the different copper exposure levels.

Firmicutes, represented by a moderately sized circle, showed a clear shift toward the CME vertex of the plot, confirming its increased abundance in the high-dose copper treatment (Figure 3). This observation aligns with the previous findings from the relative abundance analysis. Actinobacteria and Actinobacteriota, while less abundant overall, also displayed a tendency toward the CME group, suggesting that these phyla may thrive under high copper conditions. Cyanobacteria interestingly showed a bias toward the CMF group, corroborating the non-linear response to copper exposure observed earlier. The less abundant phyla, including Acidobacteriota, Gemmatimonadota, and Crenarchaeota, were positioned closer to the CME vertex, indicating their relative increases in the high-dose copper treatment (Figure 3). This trend suggests that copper exposure may create conditions favorable for these typically minor components of the gut microbiome. Unidentified Bacteria, represented by a small circle near the CME vertex, further supports the earlier observation that copper stress may select for less well-characterized bacterial taxa.

### 3.2. Beta Diversity Analysis of the Silkworm Gut Microbiome

**Figure 4:**
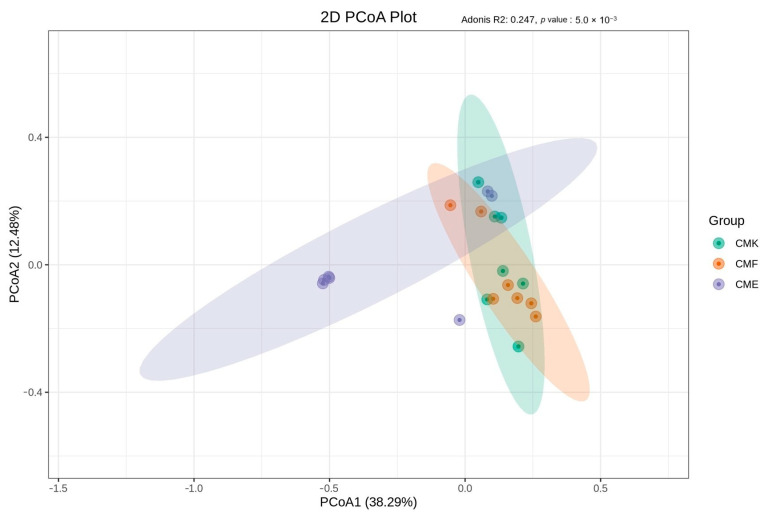
Principal Coordinate Analysis (PCoA) of the silkworm gut microbiome beta diversity based on the unweighted UniFrac distances. Each point represents an individual sample, and ellipses represent 95% confidence intervals for each group.

To elucidate the overall differences in the microbial community composition between the treatment groups, we employed multiple beta diversity analyses. The Principal Coordinate Analysis (PCoA) based on unweighted UniFrac distances revealed distinct clustering patterns between the three treatment groups (Figure 4, Table S4). The first two principal coordinates (PCoA1 and PCoA2) collectively explained 50.77% of the total variation. While the control group (CMK) and the low-dose cupric chloride group (CMF) showed considerable overlap, the high-dose cupric chloride group (CME) formed a clearly separate cluster. This separation was primarily driven by PCoA1 (38.29% of variation), indicating that high-dose copper exposure is a major factor influencing the gut microbial community structure.

**Figure 5:**
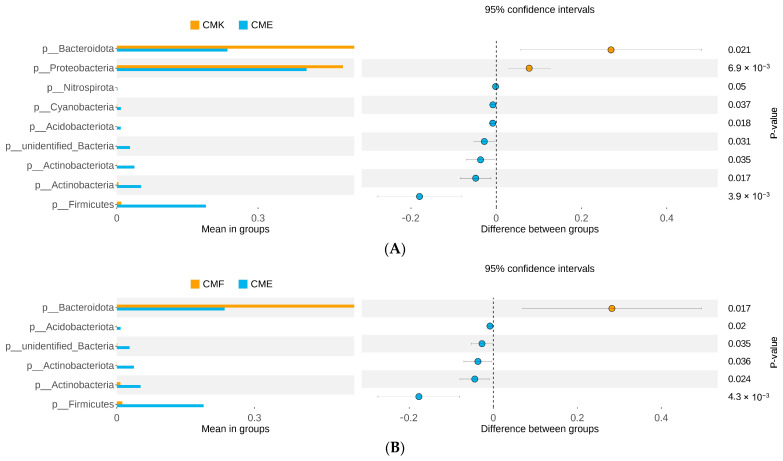
T-test-based beta diversity analysis of the ASV-level species differences between the groups in the silkworm gut microbiome under the cupric chloride exposure. (**A**) CMK vs. CME: comparison between the control group (CMK) and the high-dose cupric chloride group (CME, 8 g/kg). (**B**) CMF vs. CME: comparison between the low-dose cupric chloride group (CMF, 4 g/kg) and the high-dose cupric chloride group (CME, 8 g/kg). Each point represents a bacterial phylum. The x-axis shows the mean difference in relative abundance between the groups, while the y-axis indicates the negative logarithm of the *p*-value (−log10(*p*)). Dashed vertical lines indicate the threshold for 2-fold change in relative abundance.

To delve deeper into the specific taxonomic differences between treatment groups, we conducted a T-test-based beta diversity analysis at the ASV level (Figure 5, Table S5). This analysis revealed significant differences in the abundance of several bacterial phyla between the control (CMK) and high-dose cupric chloride (CME) groups, as well as between the low-dose (CMF) and high-dose (CME) groups. When comparing CMK with CME (Figure 5A), we observed significant changes in multiple phyla. Notably, Bacteroidota and Proteobacteria showed marked decreases in their relative abundances in the CME group (*p* = 0.021 and *p* = 6.9 × 10^−3^, respectively). Conversely, Firmicutes exhibited a significant increase in the CME group (*p* = 3.9 × 10^−3^). Other phyla that showed significant differences included Nitrospirata, Cyanobacteria, Acidobacteriota, unidentified Bacteria, and Actinobacteriota, all of which increased in relative abundance in the CME group (*p* < 0.05 for all). The comparison between the CMF and CME groups (Figure 5B, Table S6) revealed similar trends, albeit with fewer significantly different phyla. Bacteroidota again showed a significant decrease in the CME group compared with CMF (*p* = 0.017), while Firmicutes significantly increased (*p* = 4.3 × 10^−3^). Acidobacteriota, unidentified Bacteria, and Actinobacteriota also showed significant increases in the CME group (*p* < 0.05 for all). Interestingly, we did not observe significant differences in the phyla abundance between the CMK and CMF groups, suggesting that the low-dose cupric chloride treatment did not induce substantial changes in the gut microbiome composition at the phylum level. These results corroborate our earlier findings from the relative abundance analysis and beta diversity ordinations, providing statistical support for the observed shifts in the microbial community composition. The consistent decrease in Bacteroidota and increase in Firmicutes across both comparisons (CMK vs. CME and CMF vs. CME) highlighted these phyla as key responders to the high-dose cupric chloride exposure in the silkworm gut microbiome. Moreover, the lack of significant differences between CMK and CMF further supported the notion of a threshold effect, where major community shifts occurred only at higher copper concentrations.

**Figure 6:**
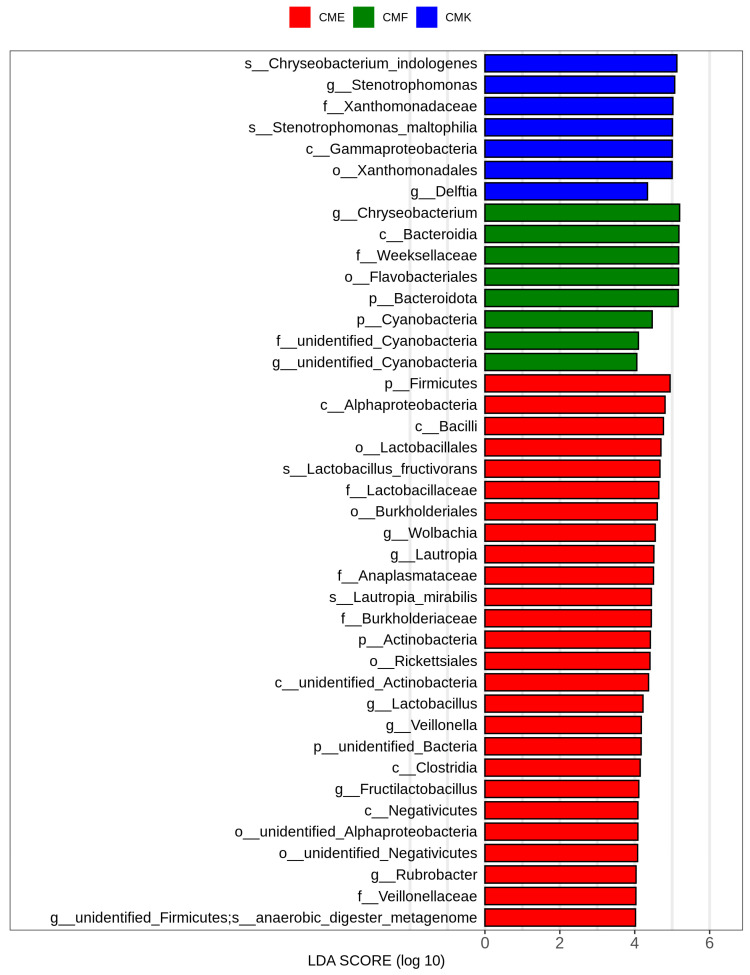
Linear discriminant analysis (LDA) effect size (LEfSe) of bacterial taxa in the silkworm gut microbiome under different cupric chloride exposure conditions. Each bar represents a specific bacterial taxon identified as a potential biomarker, with the length of the bar indicating the magnitude of the difference in abundance (LDA score > 4).

We also performed a linear discriminant analysis (LDA) effect size (LEfSe) analysis at the ASV level (Figure 6, Table S7) to identify specific bacterial taxa. This analysis revealed several taxa with LDA scores greater than 4, indicating their potential as biomarkers for different copper exposure conditions. In the control group (CMK), we observed a significant enrichment of several taxa belonging to Proteobacteria, including *Chryseobacterium indologenes*, *Stenotrophomonas*, *Xanthomonadaceae*, and *Stenotrophomonas maltophilia*. These organisms, particularly members of the Gammaproteobacteria class, appeared to be characteristic of the normal silkworm gut microbiome. The low-dose cupric chloride group (CMF) was characterized by an increased abundance of Bacteroidota, including the genus Chryseobacterium and members of the Bacteroidia class. Additionally, the CMF group showed enrichment in Cyanobacteria, including both identified and unidentified taxa at the family and genus levels. This suggests that low levels of copper exposure may create conditions favorable for these bacterial groups. The high-dose cupric chloride group (CME) displayed the most dramatic shifts in microbial composition, where numerous taxa showed significant enrichment. Notably, there was a marked increase in Firmicutes, particularly members of the Bacilli class and other Lactobacillaceae. The CME group also showed enrichment in Alphaproteobacteria, including genera such as *Wolbachia* and *Lautropia*. To our surprise, the CME group also showed enrichment in some less common or poorly characterized members, such as unidentified Actinobacteria, Negativicutes, and Firmicutes. This suggests that high copper exposure may create niches for rare or understudied bacterial groups.

**Figure 7:**
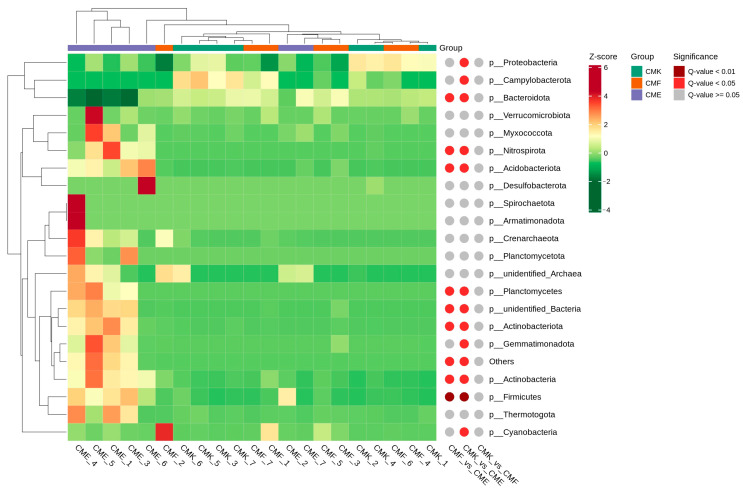
Heatmap of differential abundance analysis for the top 35 microbial taxa at the phylum level in the silkworm gut microbiome under cupric chloride exposure. The heatmap displays the relative abundance (Z-score normalized) of the top 35 microbial taxa across all samples. The rows represent individual phyla, while the columns represent individual samples. The dendrograms show hierarchical clustering of the samples (top) and phyla (left).

Using the Metastats method for the hypothesis testing of species abundance data across the groups, we were able to identify significantly different species. The heatmap in Figure 7 displays the relative abundance (Z-score normalized) of the top 35 microbial taxa across all samples at the phylum level (Figure 7). The hierarchical clustering of samples (top dendrogram) reveals a clear separation of the high-dose cupric chloride group (CME) from the control (CMK) and low-dose (CMF) groups, consistent with our previous beta diversity analyses. Several phyla showed significant differences in abundance between the treatment groups, as indicated by the colored circles to the right of the heatmap. Notably, Proteobacteria and Campylobacterota showed significant differences in the CMK vs. CME comparison (q-value < 0.05), with a generally lower abundance in the CME group. Bacteroidota exhibited significant differences in both the CMK vs. CME and CMF vs. CME comparisons, indicating a consistent response to high copper exposure. Firmicutes demonstrated significant differences across all group comparisons (q-value < 0.01), with a clear increase in abundance in the CME group. Several less abundant phyla, including Nitrospirata, Acidobacteriota, Planctomycetes, and unidentified Bacteria, showed significant differences in both the CMK vs. CME and CMF vs. CME comparisons, suggesting their sensitivity to high copper levels. Gemmatimonadota and Cyanobacteria displayed significant differences, specifically in the CMF vs. CME comparison, indicating a potential dose-dependent response to the copper exposure. The heatmap also reveals interesting patterns in the relative abundance of these phyla across samples. For instance, Firmicutes and certain Actinobacteriota showed consistently higher abundance in the CME samples, while Proteobacteria and Bacteroidota appeared more abundant in the CMK and CMF samples.

**Figure 8:**
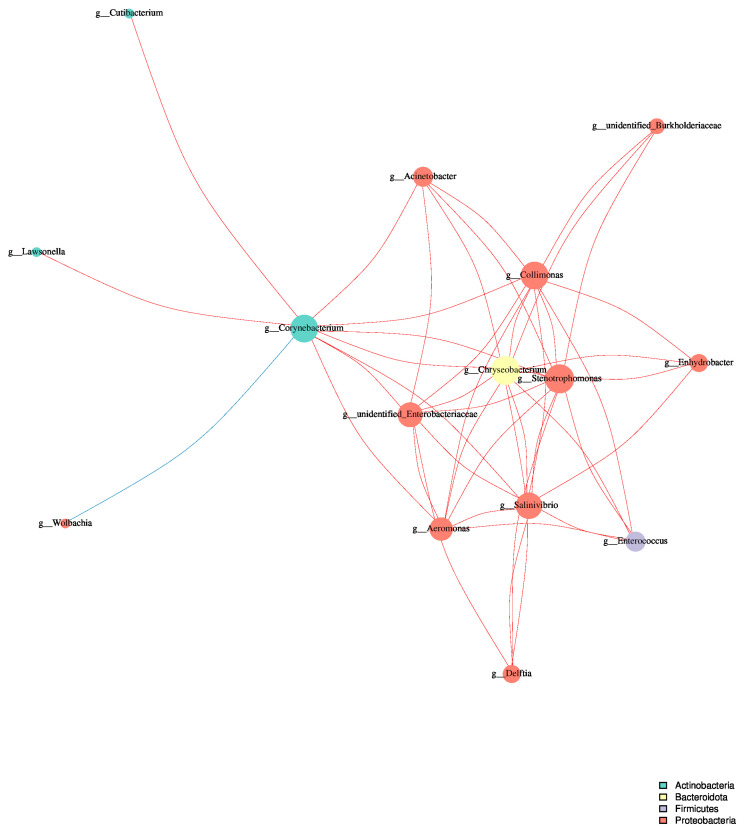
Network analysis of microbial interactions in the silkworm gut microbiome under cupric chloride exposure. Nodes represent the different genera, with the node size proportional to the degree of connectivity (number of connections). The node colors indicate different phyla, as shown in the legend. The edges between nodes represent significant correlations (|r| > 0.8) between genera, with the edge thickness proportional to the absolute correlation coefficient. Orange edges indicate positive correlations, while purple edges represent negative correlations.

Furthermore, to offer a foundation for future investigations into the mechanisms of community resilience and adaptation to metal stress, we targeted studies on specific bacterial interactions and their functional implications in the context of copper exposure. We conducted a Spearman rank correlation analysis on the top 100 most abundant genera. The resulting network diagram was constructed by applying stringent filtering criteria, including a correlation coefficient threshold of |r| > 0.8, the removal of self-connections, and the exclusion of connections for taxa with a relative abundance below 0.005% (Figure 8, Table S8). The network analysis revealed the complex interactions between various bacterial genera, providing a visual representation of the potential ecological relationships within the gut microbiome. We found that Proteobacteria (orange) and Firmicutes (purple) appeared to be the most represented phyla, consistent with their dominance in the overall community composition. Notably, some genera from Firmicutes and Proteobacteria displayed high connectivity, indicating their potential importance in maintaining the community stability or mediating the responses to copper stress. We also observed strong correlations both within and between the phyla. For instance, several Firmicutes genera showed strong positive correlations with each other, possibly indicating cooperative relationships. Conversely, some Proteobacteria genera displayed negative correlations with Firmicutes genera, suggesting potential antagonistic interactions or differential responses to copper exposure. Certain genera, represented by larger nodes with multiple connections, emerged as potential keystone taxa in the network.

### 3.3. Functional Shifts in the Silkworm Gut Microbiome Under Cupric Chloride Exposure

**Figure 9:**
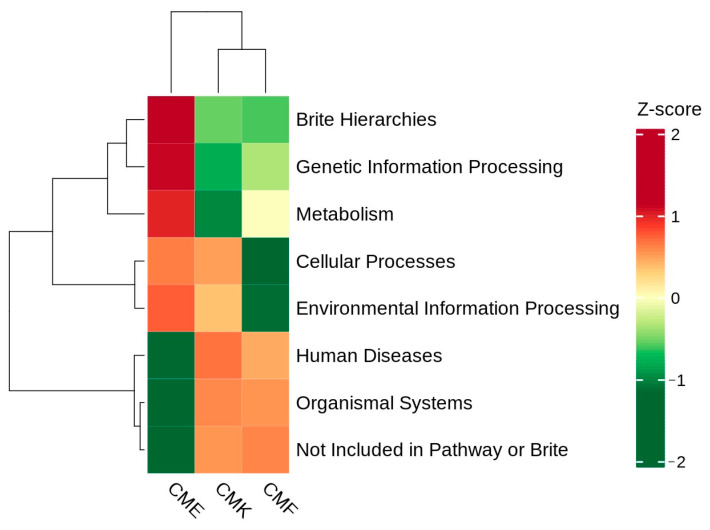
Clustered heatmap of PICRUSt2 functional annotation based on ASV data in the silkworm gut microbiome under cupric chloride exposure. Each row represents a functional category, while each column represents a sample. The color scale ranges from green (low relative abundance) to red (high relative abundance). Both the functional categories and samples were hierarchically clustered, as shown by the dendrograms on the left and top of the heatmap, respectively.

From the perspective of the functional implications of the observed taxonomic shifts in the silkworm gut microbiome under cupric chloride exposure, we performed PICRUSt2 functional annotation analysis based on ASV data (Table S9). Distinct functional profiles were revealed between the treatment groups, with clear differences between the groups (Figure 9). The hierarchical clustering of both the functional categories and samples indicates that CMK and CMF shared more similar functional profiles, while CME exhibited a notably different pattern. The Brite hierarchies and genetic information processing showed the highest relative abundance in the CME group, suggesting an upregulation of these functions in response to the high copper exposure. The metabolism-related functions appear to be relatively consistent across all the groups, with a slight increase in the CME group compared with CMK and CMF. The cellular processes and environmental information processing exhibited lower relative abundances in the CME group compared with CMK and CMF, indicating a potential downregulation of these functions under high copper stress. The human diseases, organismal systems, and functions not included in the pathway or Brite categories showed the lowest relative abundances in the CME group, suggesting a shift away from these functions in favor of more essential processes under high copper exposure. The control group (CMK) and low-dose group (CMF) showed similar functional profiles for most categories, consistent with our earlier observations of minimal community changes at the low copper concentrations. These findings suggest that high-dose cupric chloride exposure induces significant functional shifts in the silkworm gut microbiome, with a notable increase in genetic information processing and certain metabolic functions, possibly as adaptive responses to metal stress. The observed functional changes correlated with the taxonomic shifts identified earlier, particularly the increase in Firmicutes abundance in the CME group, which may have contributed to the enhanced genetic information processing and metabolic functions.

**Figure 10:**
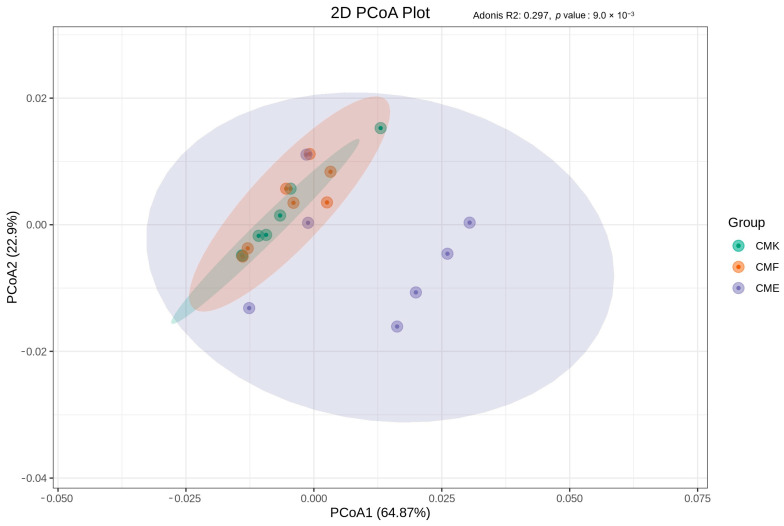
PCoA of predicted functional profiles based on the PICRUSt2 annotation of ASV data in the silkworm gut microbiome under the cupric chloride exposure. Each point represents an individual sample, and the colors indicate different treatment groups: CMK (orange), CMF (green), and CME (purple). The ellipses represent the 95% confidence intervals.

To provide insight into the overall functional similarities and differences between the samples and treatment groups, we performed a PCoA based on the PICRUSt2 functional annotation of the ASV data (Figure 10, Table S10). The PCoA plot revealed a clear separation of functional profiles between the three treatment groups. The first two principal coordinates (PCoA1 and PCoA2) explain a total of 87.83% of the variation in the data (PCoA1: 64.87%, PCoA2: 22.96%), indicating that these two axes captured the majority of the functional differences between the samples. The Adonis R^2^ value of 0.297 (*p*-value = 0.009) indicates that the treatment groups explained approximately 29.7% of the variation in the functional profiles, and this effect was statistically significant.

The CME group (high-dose) shows the most pronounced separation from the control group (CMK), while the CMF group (low-dose) occupies an intermediate position. This indicates a dose-dependent effect of cupric chloride on the microbial community function. The CME samples show a wider spread along both the PCoA axes compared with CMK and CMF, suggesting that high-dose copper exposure may lead to more variable functional profiles between individual samples. Notably, there was some overlap between the CMK and CMF clusters, indicating that low-dose copper exposure induced more subtle functional changes compared with the high-dose treatment. These results complement our previous analyses by demonstrating that the taxonomic shifts induced by the cupric chloride exposure were accompanied by significant changes in the predicted functional capabilities of the gut microbiome. The clear separation of the CME group suggests that high-dose copper exposure leads to substantial functional reorganization of the microbial community, potentially reflecting adaptive responses to metal stress. The intermediate positioning of the CMF group indicates that even a low-dose exposure can induce detectable functional changes, albeit to a lesser extent than a high-dose exposure.

**Figure 11:**
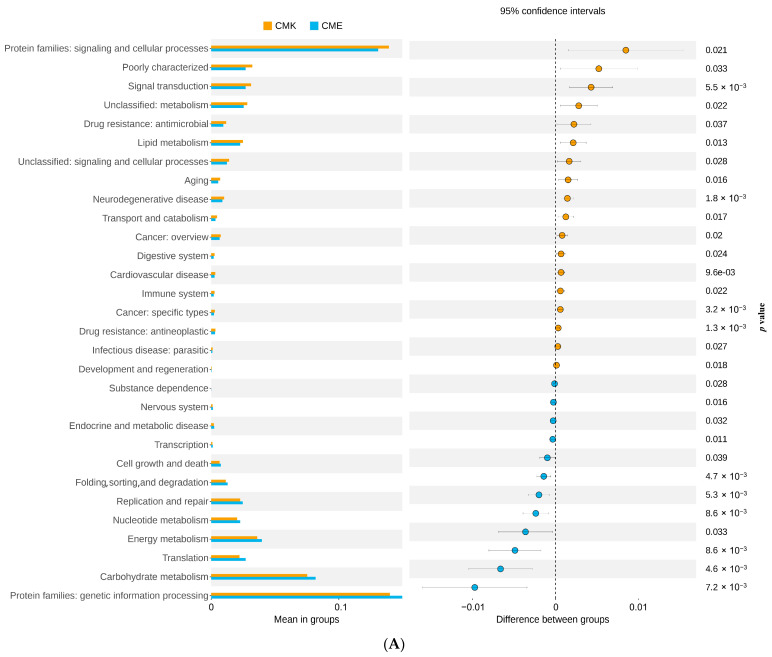

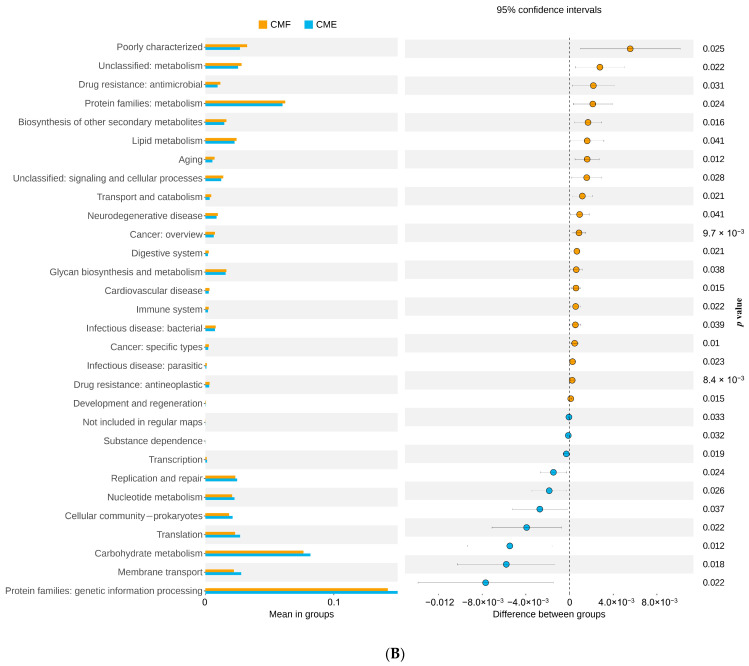
Differential KEGG pathway analysis based on PICRUSt2 predictions comparing treatment groups. (**A**) CMK vs. CME; (**B**) CMF vs. CME. The length of each bar represents the negative logarithm of the *p*-value (−log10(*p*)).

Then, we employed PICRUSt2 to predict the KEGG pathways and conducted T-test comparisons between the treatment groups (Figure 11). In the comparison between CMK and CME (Figure 11A, Table S11), we observed a marked upregulation of pathways related to cellular processes and signaling in the high-dose copper group. Notably, “Protein families: signaling and cellular processes” showed the most significant increase (*p* = 0.001), suggesting an enhanced cellular response to copper-induced stress. This was accompanied by significant increases in “Signal transduction” (*p* = 5.9 × 10^−3^) and “Unclassified: metabolism” (*p* = 0.022), indicating broad metabolic reprogramming in response to copper exposure. Intriguingly, pathways associated with “Drug resistance: antimicrobial” (*p* = 0.037) were also upregulated in CME, potentially reflecting the microbiome’s adaptive response to the antimicrobial properties of copper. The upregulation of “Lipid metabolism” (*p* = 0.013) suggests alterations in the membrane composition, possibly as a protective mechanism against copper toxicity. Conversely, several pathways showed significant downregulation in the CME group compared with CMK. These included “Carbohydrate metabolism” (*p* = 7.2 × 10^−3^), “Energy metabolism” (*p* = 8.6 × 10^−3^), and “Translation” (*p* = 8.6 × 10^−3^). This downregulation of the core metabolic processes suggests a shift from growth and proliferation toward survival and stress response in the high-copper environment. The comparison between CMF and CME (Figure 11B, Table S12) revealed similar trends, albeit with some notable differences. The upregulation of “Protein families: metabolism” (*p* = 0.014) and “Biosynthesis of other secondary metabolites” (*p* = 0.018) in CME suggests that even compared with low-dose exposure, high copper levels induce significant metabolic adaptations. The increased activity in “Unclassified: metabolism” (*p* = 0.022) further supports the notion of broad metabolic reprogramming under high copper stress. “Membrane transport” (*p* = 0.012) was significantly downregulated in CME compared with CMF, potentially indicating a strategy to limit the copper influx into bacterial cells. The downregulation of “Carbohydrate metabolism” (*p* = 0.012) and “Cellular community—prokaryotes” (*p* = 0.037) in CME compared with CMF suggests that high copper levels may impair the normal community interactions and metabolic processes, even beyond the effects seen at lower doses. Notably, the absence of significant differences between CMK and CMF in the KEGG pathway predictions implies that low-dose copper exposure does not substantially alter the functional profile of the gut microbiome. This suggests the existence of a threshold effect, where microbial communities can maintain functional homeostasis up to a certain level of copper exposure, beyond which significant adaptations are required.

**Figure 12:**
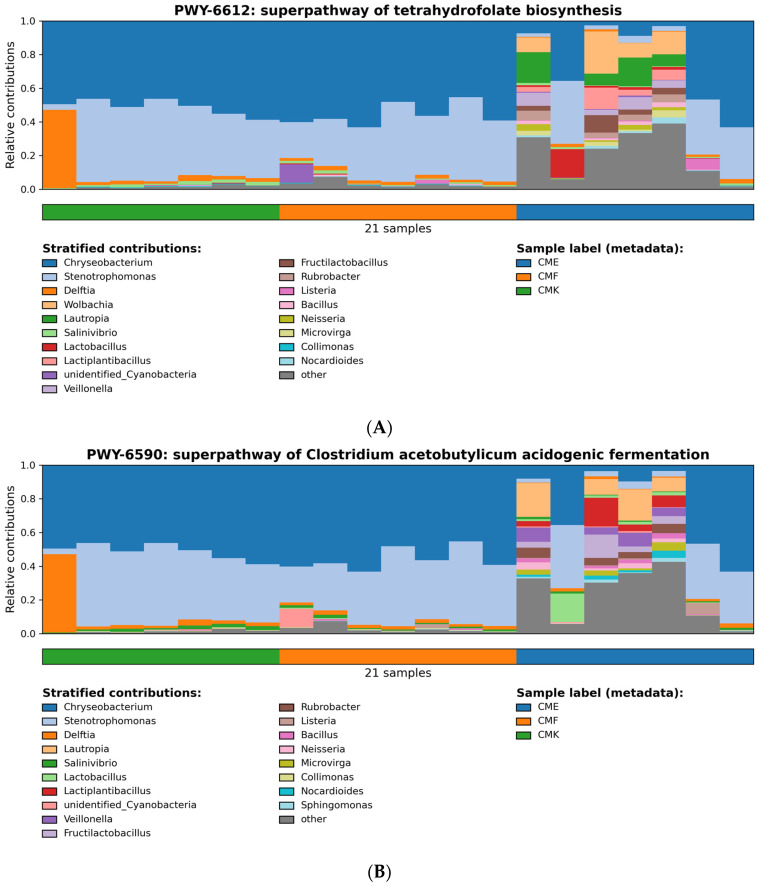
Relative contributions of the bacterial genera to the key metabolic pathways across cupric chloride treatment groups, as predicted by PICRUSt2. (**A**) Superpathway of tetrahydrofolate biosynthesis (PWY-6612). (**B**) Superpathway of Clostridium acetobutylicum acidogenic fermentation (PWY-6590). Stacked bar plots represent the relative abundances of bacterial genera that contributed to each pathway. Each bar corresponds to a sample, with samples grouped by treatment: CMK (control), CMF (low-dose cupric chloride), and CME (high-dose cupric chloride). Colors denote different bacterial genera, with the most abundant genera labeled in the legend. The y-axis shows the relative abundance of each pathway, while the x-axis represents individual samples within each treatment group.

Building upon our previous analysis, we now delve deeper into the metabolic capabilities of the silkworm gut microbiome under cupric chloride stress, focusing on two key metabolic pathways: tetrahydrofolate biosynthesis and Clostridium acetobutylicum acidogenic fermentation (Figure 12, Table S13). The superpathway of tetrahydrofolate biosynthesis (PWY-6612, Figure 12A) is crucial for one-carbon metabolism and DNA synthesis. Our analysis revealed a striking shift in the microbial contributors to this pathway across the treatment groups. In the control group (CMK), Chryseobacterium and Stenotrophomonas were the dominant contributors, collectively accounting for over 80% of the pathway’s predicted activity. However, as the cupric chloride concentration increased, we observed a marked reduction in Chryseobacterium’s contribution, particularly in the CME group. Concurrently, there was a notable increase in the relative contributions from a diverse array of genera, including *Wolbachia*, *Lautropia*, and *Lactobacillus*, especially in the CME samples. This shift suggests a potential compensatory mechanism, where copper-tolerant species assumed greater responsibility for folate biosynthesis as more sensitive species declined. The emergence of Wolbachia as a significant contributor in high-copper conditions was particularly intriguing, given its known role as an endosymbiont in many insects.

The superpathway of Clostridium acetobutylicum acidogenic fermentation (PWY-6590, Figure 12B) provides insights into the community’s capacity for anaerobic metabolism and short-chain fatty acid production. Similar to the tetrahydrofolate biosynthesis pathway, we observed a copper-dependent shift in the microbial contributors to this pathway. In the control and low-dose groups (CMK and CMF), *Chryseobacterium* and *Stenotrophomonas* again dominated the pathway’s activity. However, the high-dose group (CME) exhibited a dramatic restructuring of the contributor profile. The relative abundance of *Chryseobacterium* decreased substantially, while the contributions from genera such as *Lactiplantibacillus*, *Fructilactobacillus*, and *Veillonella* increased markedly. This shift indicates a potential adaptation of the gut microbiome toward increased fermentative metabolism under high copper stress, possibly as an alternative energy generation strategy in a more challenging environment. Interestingly, both pathways showed an increase in the “other” category in the CME group, suggesting that copper stress may be selecting for a more diverse array of minor contributors to these metabolic functions. This could represent a community-level strategy to maintain essential metabolic capabilities in the face of environmental perturbation. The consistency in patterns observed across both pathways underscores the profound impact of cupric chloride on the functional organization of the gut microbiome. The reduction in the dominance of *Chryseobacterium* and *Stenotrophomonas*, coupled with the increased contributions from a wider range of genera, pointed to a copper-induced diversification of metabolic responsibilities within the community.

## 4. Discussion

Inorganic compounds, like cupric chloride, present unique challenges due to their persistence and potential for bioaccumulation [9,12,34]. Our comprehensive analysis of the silkworm gut microbiome under cupric chloride exposure revealed profound and dose-dependent alterations in the microbial community structure and function, providing novel insights into the ecological and physiological impacts of metal stress on insect-associated microbial communities. Our findings have important implications for sericulture practices and the use of copper-based pesticides in mulberry cultivation. The observed threshold effect suggests that there is a safe range for copper application that does not significantly disrupt the silkworm gut microbiome, which is in line with many previous reports [16,17,23,35]. Based on our results, this threshold likely lies between 4 g/kg and 8 g/kg of feed. It is worth noting that typical field application rates for copper-based pesticides would likely result in residues on mulberry leaves well below this threshold, suggesting a minimal acute risk to silkworm gut microbiomes. More broadly, this study contributes to our understanding of how environmental pollutants impact host-associated microbial communities. The complex, non-linear responses observed highlight the need for comprehensive, dose-dependent analyses in ecotoxicological studies. Our results also underscore the potential of the gut microbiome as a sensitive indicator of environmental stress, which could have applications in biomonitoring and ecological risk assessment.

The observed decrease in alpha diversity metrics with increasing copper concentration aligns with the general ecological principle that environmental stress often reduces community diversity [15,36,37]. The more pronounced effects at higher copper doses (8 g/kg diet exposure) suggest a threshold-dependent response, where the resilience of the gut microbiome is overwhelmed beyond a certain level of heavy metal exposure. This non-linear response highlights the importance of considering dose-dependent effects in ecotoxicological studies and underscores the potential risks of extrapolating from low-dose experiments (4 g/kg diet exposure) to high-exposure scenarios. The taxonomic shifts observed, particularly the reduction in Bacteroidota and the concomitant increase in Firmicutes under high copper exposure, represent a significant perturbation of the normal silkworm gut microbiota. This phylum-level restructuring of the community likely reflects differential metal tolerance between bacterial taxa, as well as potential changes in the gut environment induced by copper exposure. The emergence of Firmicutes as a dominant group in high-copper conditions is particularly intriguing, given their known associations with metal resistance and stress tolerance in various environments [38,39].

Our LEfSe analysis revealed several taxa as potential biomarkers for different copper exposure conditions. The enrichment of Lactobacillus and other Firmicutes in the high-dose group corroborates their potential role in metal stress adaptation [40,41,42]. Conversely, the reduction in certain Proteobacteria and Bacteroidota taxa in high-copper conditions suggests their sensitivity to metal stress. The Metastats analysis further supported these findings, highlighting significant differences in the phylum-level abundances between the treatment groups. The consistent response of certain phyla, such as Firmicutes and Bacteroidota, across multiple comparisons underscores their importance in the community’s response to copper stress [41,42,43]. The identification of less abundant phyla showing significant changes, such as Nitrospirata and Acidobacteriota, points to the complex and far-reaching effects of copper exposure on the gut microbiome.

The observed shift in the relative abundance of Bacteroidota and Firmicutes under cupric chloride exposure aligns with findings from studies on other insect species exposed to various environmental stressors. For instance, it is reported the presence of copper and chlorpyrifos was strongly positively correlated with the abundance of genera within the Proteobacteria and Firmicutes phyla [44]. Studies on black soldier fly (*Hermetia illucens*) showed that exposure to neonicotinoid insecticides can induce comparable alterations in the Bacteroidota-to-Firmicutes ratio, suggesting a conserved response pattern across different insect orders [45]. This consistent pattern may reflect the differential metal tolerance mechanisms between these bacterial phyla, with Firmicutes generally possessing more robust stress response systems, including enhanced metal binding proteins and efflux pumps [46].

The consequences of such microbiota alterations extend beyond the immediate changes in the community composition. The alternation of Bacteroidota, which typically play crucial roles in polysaccharide metabolism and immune system modulation, may affect the host’s ability to efficiently process dietary components and maintain immune homeostasis [47]. Specifically, in lepidopteran insects, a Bacteroidota decrease was shown to enhance the intestinal barrier function and regulate the local immune responses [48,49]. Conversely, the increased abundance of Firmicutes, while potentially conferring enhanced stress tolerance, may alter the metabolic profile of the gut environment. Previous studies demonstrated that Firmicutes dominance can lead to changes in the energy harvest efficiency and the production of secondary metabolites that influence host physiology [50,51]. In the context of sericulture, the alternation of gut-microbiota-mediated effects could have cascading impacts on silk production, as both the nutrient absorption efficiency and immune status are known to influence the silk protein synthesis and fiber quality [52,53].

Furthermore, the functional predictions derived from our taxonomic data using PICRUSt2 indicate significant shifts in the metabolic and regulatory capabilities of the gut microbiome under high copper exposure. The upregulation of genetic information processing and certain metabolic pathways in the high-dose group suggests a reallocation of resources toward essential cellular functions and stress response mechanisms. This functional restructuring may represent an adaptive strategy to cope with the physiological challenges posed by elevated copper levels [12,54]. The apparent resilience of the gut microbiome to low-dose copper exposure, as evidenced by the similarity between control and low-dose groups in both taxonomic and functional analyses, is noteworthy. This observation suggests that the silkworm gut microbiome possesses inherent buffering capacity against moderate metal stress, which may be an evolutionary adaptation to variable environmental conditions. It is important to note that our low-dose treatment of 4 g/kg feed is already five times higher than the typical oral LD50 for many mammalian species, indicating a substantial tolerance in silkworms [15,23,55]. However, the dramatic shifts observed at high doses (8 g/kg) indicate that this resilience has limits, beyond which community structure and function are significantly altered.

In summary, our study revealed the complex and dose-dependent impacts of cupric chloride exposure on the silkworm gut microbiome, providing a foundation for understanding the ecological and physiological consequences of metal stress in insect–microbe symbioses. These findings not only inform agricultural practices in sericulture but also contribute to the broader field of environmental microbiology and host–microbe interactions under anthropogenic stress. The observed resilience at lower doses, coupled with the dramatic community shifts at higher concentrations, underscores the delicate balance between tolerance and disruption in microbial ecosystems. This research highlights the need for nuanced approaches in assessing the environmental impact of agricultural chemicals, considering both direct effects on target organisms and indirect effects mediated through host-associated microbiomes.

## 5. Conclusions

Our comprehensive investigation demonstrated that copper exposure induced a significant, dose-dependent restructuring of the silkworm gut microbial community, characterized by reduced alpha diversity and shifts in taxonomic composition. A high-concentration copper diet of 8 g/kg led to a dramatic phylum-level reorganization, with a notable decrease in Bacteroidota and a concomitant increase in Firmicutes, suggesting a differential metal tolerance between the major bacterial groups. Functional predictions indicate a reallocation of microbial metabolic capabilities in response to the high copper exposure, potentially reflecting adaptive strategies for coping with metal-induced stress. The gut microbiome exhibited resilience to 4 g/kg low-dose copper diet exposure, suggesting an evolved tolerance to moderate metal stress, but underwent significant perturbation beyond a critical threshold.

## Figures and Tables

**Table 1:** Alpha diversity indices of silkworm gut microbiome under different cupric chloride exposure conditions.

Group	Observed_ASV	Shannon	Simpson	Chao1	ACE	Goods_Coverage	PD_Whole_Tree
CMK	90.714	1.121	0.567	96.178	96.08	1	19.02
CMF	82.571	1.311	0.598	83.934	83.648	1	18.033
CME	262.429	3.47	0.839	265.043	265.124	1	40.821

Note: Values are presented as the mean ± standard error (*n* = 7). Different lowercase letters within the same column indicate significant differences between groups (*p* < 0.05, one-way ANOVA followed by Tukey’s HSD test). ACE: Abundance-based Coverage Estimato.

## Data Availability

The raw 16S ASV count table can be accessed publicly on Figshare under https://doi.org/10.6084/m9.figshare.27308724.

## Supplementary Materials

The following supporting information can be downloaded at: https://www.mdpi.com/article/10.3390/ani14243634/s1. Table S1. Raw ASV count table for all sequenced samples. Table S2. Relative abundance of taxonomic groups at the phylum level. Table S3. Relative abundance of ASVs across all samples. Table S4. Principal Coordinate Analysis (PCoA) scores based on unweighted UniFrac distances. Table S5. Results of t-test analysis that compared ASV abundance between CMK and CME groups. Table S6. Results of t-test analysis that compared ASV abundance between CMF and CME groups. Table S7. ASV table with corresponding LEfSe values. Table S8. SparCC covariance matrix for genus-level correlations. Table S9. PICRUSt2 predicted KEGG pathways at Level 2. Table S10. PCoA scores based on Bray–Curtis distances of PICRUSt2 KEGG pathway predictions. Table S11. Differential abundance analysis of PICRUSt2 KEGG pathways (Level 2) between CMK and CME groups. Table S12. Differential abundance analysis of PICRUSt2 KEGG pathways (Level 2) between CMF and CME groups. Table S13. Relative contributions of bacterial genera to key metabolic pathways.
